# Protein Design Enters the Artificial Intelligence Era: Foundations, Tools, and Emerging Paradigms

**DOI:** 10.34133/csbj.0105

**Published:** 2026-06-01

**Authors:** Yanlin Mi, Arpit Shukla, Mark Tangney, Sabin Tabirca, Venkata VB Yallapragada

**Affiliations:** ^1^ School of Computer Science and Information Technology, University College Cork, College Road, T12K8AF, Cork, Ireland.; ^2^ Centre for Research Training in Artificial Intelligence, University College Cork, College Road, T12K8AF, Cork, Ireland.; ^3^ School of Software and AI, Yunnan University, South Waihuan East Road, 650504, Kunming, China.; ^4^ Cancer Research @UCC, College of Medicine and Health, University College Cork, T12 K8AF, Cork, Ireland.; ^5^ APC Microbiome Ireland, University College Cork, T12 YT20, Cork, Ireland.; ^6^ School of Biotechnology & Bioengineering, Institute of Advanced Research, Koba Institutional Area, Gandhinagar, 382426, Gujarat, India.; ^7^ Faculty of Mathematics and Informatics, Transylvania University of Brasov, Bulevardul Eroilor 29, 500036, Brasov, Romania.; ^8^ Centre for Advanced Photonics and Process Analytics, Munster Technological University, Rossa Ave, T12P928, Cork, Ireland.

## Abstract

Artificial intelligence (AI) has transformed protein engineering by leveraging deep learning, protein language models, and knowledge graphs to decode relationships between sequence, structure, and function. Models like AlphaFold2 achieve near-experimental accuracy in structure prediction, while transformer-based language models facilitate de novo sequence design under functional constraints. AI enhances therapeutic protein engineering, enzyme catalysis, and synthetic biology, accelerating the transition from in silico design to experimental validation. These advances accelerate experimental validation across healthcare and industrial biotechnology. Despite algorithmic successes, challenges remain in model interpretability, training data biases, and experimental validation rates. This review examines the computational methodologies shaping protein design, benchmarking metrics, and the integration of machine learning with experimental pipelines.

## Highlights

• **AI technologies revolutionizing protein design:** The review analyzes 4 key AI technologies transforming protein engineering, highlighting their technical implementations, strengths, and limitations.
• **Commercial breakthroughs across sectors:** The review emphasizes the substantial commercial impact of AI in therapeutics, food technology, and industrial biotechnology, showcasing innovations like AI-accelerated vaccine development and next-generation plant-based proteins.
• **Critical insights into AI-experimental integration:** The paper highlights emerging trends such as hybrid physical–AI approaches, which promise to bridge the gap between computational predictions and experimental validation, addressing key challenges in protein design.

## Graphical Abstract

**Figure:**
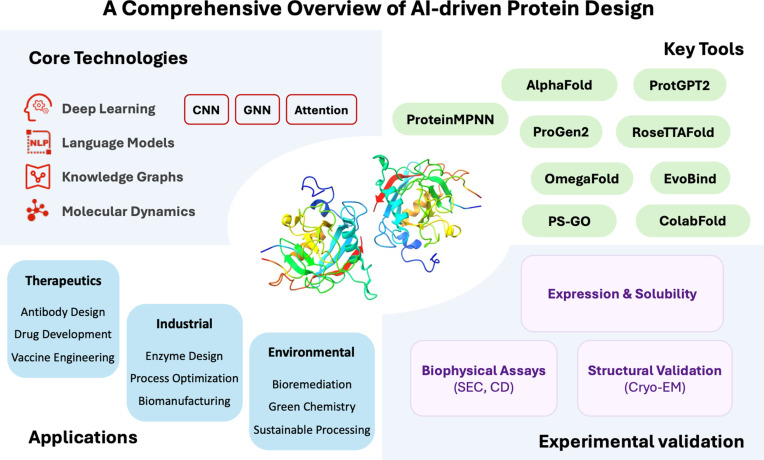

## The AI-Driven Protein Engineering Landscape

The demand across pharmaceutical, biotechnology, and industrial sectors for precisely engineered proteins drives rapid expansion in computational biology. The field differentiates between engineered proteins—which involve targeted modifications to existing natural scaffolds to optimize parameters such as thermal stability or catalytic turnover—and de novo designer proteins. De novo design represents the synthesis of entirely novel topologies and sequences that do not exist in the evolutionary record, constructed fundamentally from biophysical principles or generative latent spaces. The global protein engineering market size accounted for USD 4.35 billion in 2024, grew to USD 5.09 billion in 2025, and is expected to be worth around USD 20.86 billion by 2034, poised to grow at a compound annual growth rate of 16.97% between 2024 and 2034 [1]. This expansion is driven by increasing demands across pharmaceutical, biotechnology, and industrial sectors for precisely engineered proteins with enhanced or novel functions [2]. Engineered proteins, which encompass both rationally modified natural proteins and de novo designed proteins, represent a cornerstone of modern biotechnology. While engineered proteins typically involve modifications to existing protein scaffolds to enhance specific properties, designer proteins represent a more ambitious frontier where entirely novel protein structures and functions are created from scratch. This distinction is particularly relevant as artificial intelligence (AI) technologies increasingly enable both the optimization of natural proteins and the creation of unprecedented protein architectures.

Proteins are nature’s molecular machines, orchestrating virtually every biological process through their remarkable diversity of functions. From catalyzing complex chemical reactions to transmitting cellular signals and providing structural support, proteins are fundamental to life itself [3–5]. Their functionality derives from precisely folded 3-dimensional structures, determined by specific sequences of amino acids. This intricate relationship between sequence, structure, and function has long fascinated scientists and engineers, driving efforts to design proteins with novel and enhanced capabilities [6–8].

The significance of protein design extends far beyond academic interest. In medicine, engineered proteins form the basis of numerous therapeutics, from insulin analogs to monoclonal antibodies. AI-driven companies like AbCellera successfully developed COVID-19 antibody treatment (bamlanivimab) and are accelerating this field [9]. In industrial biotechnology, engineered enzymes designed by companies such as Zymergen and Ginkgo Bioworks catalyze processes from biofuel production to waste treatment [10]. Recent achievements highlight the field’s potential: Researchers have created antibody nanocages that enhance therapeutic delivery [11,12], developed novel protein assemblies with programmable geometries [13,14], and engineered protein-based molecular machines with sophisticated functions [13].

The commercial landscape for AI in protein design is expanding at an unprecedented rate, underscored by a surge in high-value partnerships between AI-native biotechnology companies and established pharmaceutical giants. As shown in Table 1, these collaborations, often involving potential milestone payments exceeding a billion dollars, highlight the industry’s conviction in AI’s potential to break through long-standing bottlenecks in drug discovery [15,16]. The following Table 1 provides an overview of some of the most important recent partnerships, moving beyond a simple market summary to analyze the specific AI applications being deployed and the core technical challenges these collaborations aim to solve. Analysis of these partnerships reveals several key trends. First, the financial commitments are substantial, indicating that AI-driven design is now a central pillar of pharmaceutical research and development strategy. Second, the scope of these deals is broad, targeting not only antibodies but also the historically challenging domain of small-molecule design (as seen in the Isomorphic Labs collaborations). Third, the focus is increasingly on generative approaches, creating novel molecular structures rather than simply screening existing ones. However, the “Key technical challenge” column underscores a critical reality: While AI models can generate promising candidates, ensuring these digital designs translate into safe, effective, and manufacturable drugs remains the primary hurdle for the entire field.

**Table 1.** Overview of recent major AI-driven drug discovery partnerships

Date (announced)	AI company	Pharmaceutical partner	Therapeutic focus	Maximum potential value (USD)	AI application	Key technical challenge
Jan 2024	Isomorphic Labs	Eli Lilly	Multiple small molecules	$1.7 B	Structure-based generative design of novel small-molecule drugs.	Modeling complex protein–ligand binding energetics and predicting off-target interaction networks
Jan 2024	Isomorphic Labs	Novartis	Multiple small molecules	$1.2 B	Predicting protein structures and designing novel chemical entities.	Integrating structural predictions with multiparameter optimization (MPO) for pharmacokinetic and toxicity profiling
Jan 2024	Generate Biomedicines	Amgen	Multiple therapeutic areas	$1.9 B	Generative AI platform (Chroma) to create novel protein-based therapeutics.	Optimizing multidomain folding pathways and minimizing sequence immunogenicity in de novo constructs
May 2024	Absci	Merck	Oncology	$610 M	De novo design of therapeutic antibodies against specified targets.	Modeling complementarity-determining region (CDR) loop flexibility and optimizing antigen-specific interface packing
Nov 2023	BioMap	Sanofi	Multiple therapeutic areas	$1 B	Modeling the immune system to discover and design novel biologics.	Integrating multiomics knowledge graphs to decode allosteric modulation and identify novel therapeutic interaction nodes

AI, artificial intelligence

Traditional approaches to protein design have relied heavily on rational design and directed evolution. Rational design attempts to predict beneficial mutations based on structural knowledge and biochemical principles, while directed evolution mimics natural selection in the laboratory to optimize protein properties. These methods, while powerful, face important limitations. The complexity of protein folding, the vast sequence space to explore (20^100^ possible sequences for a modest 100-amino-acid protein), and the intricate relationships between sequence, structure, and function have historically made protein design a challenging endeavor [17,18]. To contextualize the integration of AI, it is necessary to trace the methodological evolution of protein design. The field rests on the foundation of Anfinsen’s dogma, which posits that a protein’s amino acid sequence determines its 3-dimensional structure. Early computational protein design (CPD) frameworks, such as Rosetta, operationalized this principle using physics-based energy functions and heuristic sampling algorithms. These classical methods search conformational space to identify sequences that minimize free energy for a target structure. While highly successful in generating stable folds, traditional CPD is computationally expensive and struggles with complex multistate dynamic modeling. The recent integration of AI does not replace these biophysical principles; rather, it circumvents the computationally prohibitive energy-landscape search by learning evolutionary and spatial patterns directly from existing protein databases.

The emergence of AI has initiated a fundamental transformation in protein design methodology. This revolution began with improvements in protein structure prediction, exemplified by AlphaFold2’s breakthrough in achieving near-experimental accuracy [19,20]. This advance has been complemented by developments in protein language models and deep learning approaches that can generate novel protein sequences with desired properties [21,22]. The impact of these technologies extends beyond mere computational improvements; they have fundamentally changed how we approach the protein design problem.

AI methods are starting to advance protein design through several transformative capabilities. Deep learning approaches enable rapid exploration of vast sequence spaces through efficient sampling algorithms that learn from existing protein structures and sequences. These methods allow direct optimization of protein properties through differentiable models that can be trained end-to-end. Additionally, AI facilitates the integration of multiple data modalities, including sequence, structure, and functional information, providing a more comprehensive approach to protein design. The technology also enables the automated design of complex protein architectures previously considered impossible to engineer [20,21,23].

Consider the example of de novo protein design, where AI has enabled the creation of proteins with entirely novel folds. Traditional methods typically began with known protein structures and made incremental modifications. In contrast, AI approaches can generate completely new protein architectures by learning the fundamental principles of protein folding from existing structures. This capability was demonstrated in recent work where researchers used deep learning to create protein structures with unprecedented geometric complexity, including protein rings, cages, and lattices with precise specifications [13,24–26].

**Fig. 1.**
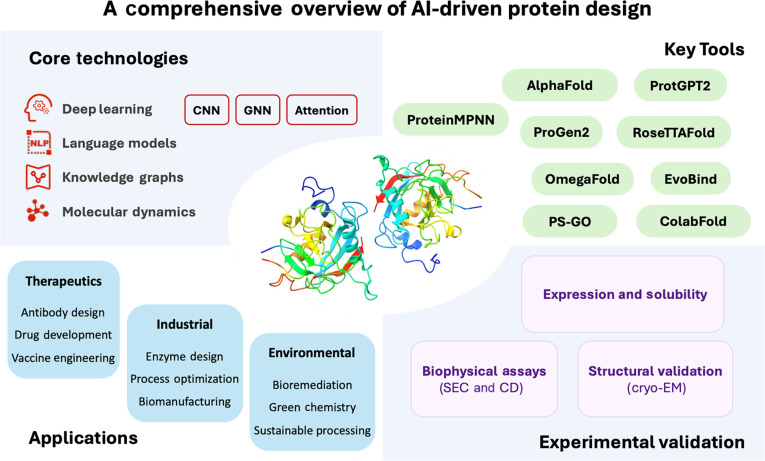
A comprehensive overview of artificial intelligence (AI)-driven protein design. The diagram is organized into several key sections: On the top left, it lists core technologies including deep learning (with convolutional neural network [CNN], graph neural network [GNN], and Attention mechanisms), language models, knowledge graphs, and molecular dynamics; at the center, it features a colorful 3-dimensional (3D) protein structure illustration; the top right shows key tools including AlphaFold and RoseTTAFold. The lower left section outlines 3 major application domains (therapeutics, industrial, and environmental) with their specific use cases. Additionally, the market impact is highlighted in the lower right. cryo-EM, cryo-electron microscopy; CD, circular dichroism; SEC, size exclusion chromatography.

In summary, the confluence of AI technologies with protein design has ushered in a new era of possibilities in protein engineering. From enhanced structure prediction capabilities to sophisticated sequence generation models, AI methods have started to fundamentally alter both the scope and efficiency of protein design. These advances have enabled unprecedented achievements in therapeutic development, industrial applications, and fundamental protein science. As shown in Fig. 1, the following sections delve into the key AI technologies driving these advances, examining their technical implementations, practical applications, market impact and the challenges that lie ahead. We begin by exploring the core AI methodologies that form the foundation of modern protein design, including deep learning architectures, protein language models, and knowledge graph frameworks, before proceeding to discuss their practical implementations and impact across various domains.

## Key AI Technologies in Protein Design

The transition from traditional computational methods to AI-driven approaches has been enabled by advances in 4 key areas: deep learning architectures, protein language models, knowledge graph technologies, and the integration of molecular dynamics simulations. Understanding these foundations is crucial for appreciating the current capabilities and future potential of AI in protein design. Each of these technological pillars contributes unique capabilities to the protein design process, from structure prediction and sequence optimization to dynamic behavior analysis and knowledge integration. Contemporary AI methodologies in protein design partition into distinct operational frameworks. Discriminative models (e.g., AlphaFold2) map sequences to structural coordinates, whereas generative models (e.g., ProteinMPNN and RFdiffusion) solve the inverse problem, mapping desired topologies or functions to viable sequences. These approaches differ fundamentally in representational assumptions: Language models rely on 1-dimensional sequence data, assuming evolutionary covariation encodes structure, whereas graph-based and diffusion models explicitly condition on 3-dimensional (3D) spatial representations. A critical limitation across these data-driven approaches is training data bias. Language models suffer from homology bias, frequently failing to generalize to regions of sequence space unrepresented in UniProt. Similarly, discriminative structure predictors risk dataset leakage, where models memorize local structural motifs from the Protein Data Bank rather than learning generalized folding biophysics.

### Deep learning architectures

The evolution of deep learning architectures in protein design has been marked by increasingly complex approaches to handling the complex hierarchical nature of protein structures [27–30]. The implementation of these architectures has revealed several key innovations in network design and training methodologies, particularly in the realm of convolutional neural networks (CNNs) and their adaptations for protein structure analysis [31].

Advanced deep learning architectures for protein design rely on specific mathematical modifications to process 3-dimensional structural data accurately. In graph neural networks (GNNs), representing proteins requires maintaining geometric consistencies. Modern implementations utilize SE(3)-equivariant networks, which ensure that translation and rotation operations on the input 3D coordinates result in corresponding transformations in the output vector space. This equivariance allows the network to learn structural motifs independent of the global coordinate frame. Furthermore, attention mechanisms have evolved to directly process spatial coordinates. Architectures such as AlphaFold2 employ Invariant Point Attention. Instead of computing attention solely from 1-dimensional sequence embeddings, Invariant Point Attention represents each residue as a local 3D frame containing rotation and translation data. It calculates attention weights using the invariant distances between these local frames. This mechanism guarantees that structural representations remain invariant to global rotations and translations, allowing the model to capture long-range spatial dependencies and side-chain packing geometry accurately.

**Fig. 2.**
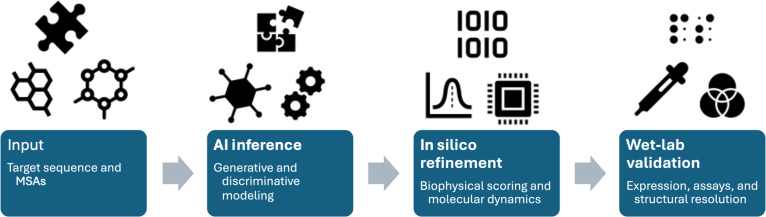
Methodological workflow for artificial intelligence (AI)-driven protein design. The pipeline begins with the compilation of target amino acid sequences and multiple sequence alignments (MSAs). During the AI inference phase, discriminative models predict structural coordinates, while generative models synthesize novel sequence backbones. Promising candidates proceed to in silico refinement, where physics-based energy scoring and molecular dynamics evaluate thermodynamic stability. The workflow concludes with wet-lab validation, providing empirical confirmation through protein expression, biophysical assays (e.g., size exclusion chromatography), and structural resolution (e.g., x-ray crystallography or cryo-electron microscopy [cryo-EM]).

Modern CNN architectures have evolved to incorporate dilated convolutions specifically designed for protein structure analysis [31,32]. As shown in Fig. 2A, these networks employ a complex multiscale approach, utilizing various dilation rates simultaneously to capture structural patterns at different spatial scales. A typical implementation employs dilation rates of 1, 2, 4, and 8, enabling the network to process both local and long-range structural patterns effectively. The architecture is further enhanced by residual connections between dilated convolutional layers, which maintain robust gradient flow during training while processing extended structural patterns [29,33,34].

Advanced CNN architectures designed specifically for protein analysis incorporate multiple parallel convolutional streams, each dedicated to processing distinct structural features. One stream focuses on backbone angle predictions, while others simultaneously process side-chain orientations and contact maps. These streams are complemented by specialized pooling operations that successfully reduce computational complexity while preserving critical structural information. The architecture’s effectiveness is further enhanced by attention-gated convolutions, which dynamically adjust filter weights based on the local structural context of each protein region [35–37].

GNNs in protein design have undergone substantial evolution, now incorporating complex edge features and message-passing schemes [38,39]. Contemporary implementations utilize comprehensive edge features that encode both covalent and noncovalent interactions. These features capture the full spectrum of molecular interactions, including hydrogen bonds, hydrophobic interactions, and electrostatic forces. As shown in Fig. 2B, the networks implement multiple rounds of message passing with learned update functions, allowing for sophisticated information propagation across the protein structure. The architecture is completed by hierarchical graph pooling operations that maintain the essential aspects of protein topology while reducing computational complexity [26,40].

A notable breakthrough in GNN architecture has been the development of equivariant GNNs. These networks maintain geometric invariances crucial for protein structure prediction through the implementation of SO(3)-equivariant layers. These specialized layers process geometric features while preserving rotational invariance, a property that has proven essential for accurate structure prediction and validation [41–43].

### Diffusion-based generative models

Diffusion-based generative models operate via a predefined Markov chain that progressively corrupts data with Gaussian noise, followed by a learned reverse denoising process. Utilizing denoising score matching, these models learn the gradient of the data distribution, enabling the recovery of coherent structures from random noise. Methods partition into 2 distinct modalities: continuous and discrete. Continuous models, such as RFdiffusion, operate in 3D coordinate space, diffusing the translational and rotational coordinates of the protein backbone. These models support spatial conditioning mechanisms, such as motif scaffolding, where the denoising trajectory is constrained to preserve a predefined active site geometry. Conversely, discrete diffusion models, such as EvoDiff, execute the forward and reverse processes directly over categorical sequence space without structural priors. Compared to autoregressive models (which generate sequences directionally) or generative adversarial networks (which suffer from mode collapse), diffusion models exhibit superior sampling diversity and structural controllability, albeit at a higher computational inference cost.

Contemporary diffusion models implement fundamentally distinct design philosophies. RFdiffusion operates on continuous spatial coordinates, applying a denoising process conditioned on physical constraints to generate structurally viable backbones. It utilizes a trained network to recover specific tertiary geometries, maintaining high structural fidelity. Chroma similarly utilizes a score-based generative model with GNNs but introduces programmable constraints, enabling scalable simulations of complex symmetries and dynamic folding states [44]. Conversely, EvoDiff circumvents spatial requirements by executing a discrete diffusion process directly over sequence space. This sequence-only approach enables the generation of intrinsically disordered proteins and novel topologies independent of established structural templates [44,45]. Table 2 summarizes their primary applications, representative tools, and inherent strengths and limitations. This comparative overview highlights the shift from discriminative models used for analysis to generative models that are now capable of creating novel proteins from scratch.

**Table 2.** Comparative overview of key deep learning architectures in protein design

Model architecture	Representative tool(s)	Conditioning mechanisms	Primary protein design application(s)	Strengths	Limitations
Graph neural networks (GNNs)	ProteinMPNN	Fixed 3D backbone coordinates; symmetric constraints.	Designing sequences that fold into a predefined backbone structure.	Excels at capturing the 3D local environment of residues for high-fidelity sequence recovery.	Requires a given backbone; cannot generate novel folds.
Language models (LMs)	ESM-2, ProGen2	Functional tags; evolutionary sequence context; zero-shot prompting.	Generating novel sequences based on learned evolutionary patterns.	Learns functional patterns from vast sequence data without needing structures.	Indirect control over 3D structure; generated sequences may not fold correctly.
Diffusion models	RFdiffusion	Motif scaffolding; partial structures; target binding interfaces.	Generating novel protein backbones from scratch, with or without functional constraints (motif scaffolding).	Generates entirely new folds not seen in nature; can be precisely conditioned.	Computationally intensive; ensuring biological function remains a challenge.

3D, 3-dimensional

Recent innovations in attention mechanisms have substantially improved protein design capabilities [45]. Advanced implementations now utilize axial attention mechanisms that separately process spatial and channel dimensions, allowing for more efficient and effective feature extraction. These systems are complemented by multihead attention mechanisms, where different heads specialize in detecting various types of structural patterns. The effectiveness of these attention mechanisms is further enhanced by gradient-guided attention systems, which utilize backpropagated gradients to focus computational resources on functionally important regions of the protein structure [46,47].

These sophisticated attention mechanisms have been particularly revolutionary in capturing the complex dependencies in protein structures. As shown in Fig. 2C, position-specific attention weights now incorporate both sequence and structural context, enabling the model to learn subtle relationships between distant residues that may be spatially proximate in the folded structure. This has proven especially valuable for understanding allosteric effects and long-range interactions that influence protein function [48–50].

The integration of structure-aware attention has led to substantial improvements in modeling protein conformational changes. By incorporating distance matrices and contact maps into the attention computation, these mechanisms can better capture the physical constraints and dynamic nature of protein structures. This has enabled more accurate prediction of protein flexibility, conformational switches, and interaction interfaces [51,52].

Furthermore, recent implementations have introduced hierarchical attention schemes that operate at multiple structural levels—from local backbone geometry to domain-level organization. This multiscale approach allows the model to simultaneously consider both fine-grained atomic interactions and larger-scale architectural features that determine protein stability and function. The attention weights at each level are dynamically adjusted based on the specific design task, whether it is optimizing binding interfaces, engineering allosteric regulation, or designing novel folds [53–55].

### Protein language models

**Fig. 3.**
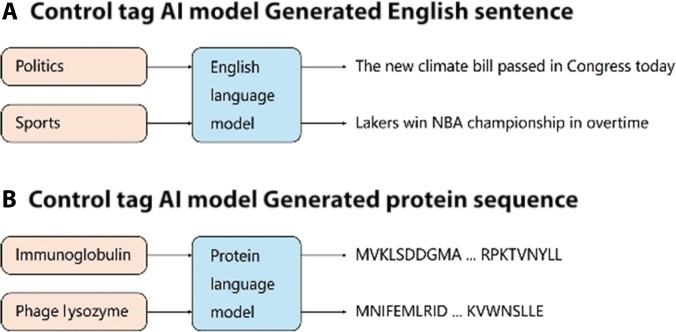
The conceptual parallel between natural language models and protein language models. (A) It demonstrates a conventional English language model that generates domain-specific sentences based on control tags (e.g., “Politics” or “Sports”). (B) It shows an analogous protein language model that generates amino acid sequences conditioned on protein family tags (e.g., “Immunoglobulin” or “Phage Lysozyme”). This comparison highlights how the fundamental principles of large language models can be adapted to biological sequence generation: Just as language models learn to produce contextually appropriate text based on input prompts, protein language models learn to generate biochemically viable amino acid sequences based on protein family specifications. The architectural similarity between these models suggests that the underlying patterns in protein sequences can be captured and generated using similar computational frameworks as those used for natural language processing.

As shown in Fig. 3, by analogy with the English language model, we can easily understand the protein language model. Modern protein language models have achieved remarkable advances through sophisticated architectural innovations and training strategies [56]. The training process has evolved to incorporate multiple complementary objectives that enhance the models’ understanding of protein structure and function. These objectives include structure-aware masked token prediction, which strategically masks portions of protein sequences to force the model to learn structural patterns. This is complemented by domain-adapted next sentence prediction tasks that help the model understand the relationships between different protein domains. Additionally, contrastive learning between sequence and structure representations has proven particularly effective in developing rich, informative protein embeddings [8,32].

The training methodology has been refined to overcome computational limitations while maximizing model performance. Modern approaches implement gradient accumulation across multiple graphics processing units, enabling the use of larger effective batch sizes that improve training stability and model convergence. This is combined with mixed-precision training techniques that reduce memory requirements while maintaining numerical stability. The training process typically follows a curriculum learning approach, beginning with shorter sequences and progressively increasing sequence length as training proceeds, allowing the model to build up complexity gradually [57–59].

Architectural innovations have substantially enhanced model performance. Recent models incorporate relative positional encodings that more accurately capture local structural relationships within proteins. These encodings are complemented by sparse attention patterns that focus computational resources on evolutionarily related positions, improving the model’s ability to capture meaningful sequence relationships. The architecture is further enhanced by specialized embedding layers that encode detailed amino acid chemical properties, providing the model with rich input representations that facilitate the learning of structure–function relationships [32,56,60,61].

### Knowledge graph technologies

The implementation of knowledge graph technologies in protein design represents a sophisticated approach to integrating and reasoning about complex biological information [10]. Modern protein knowledge graphs have evolved to incorporate multiple layers of biological information through advanced construction techniques and reasoning mechanisms. The graph construction process has been revolutionized by automated extraction systems that utilize specialized biomedical natural language processing algorithms to mine protein–protein interactions from scientific literature. These systems achieve high precision through context-aware entity recognition and relationship extraction, substantially reducing the manual curation burden while maintaining data quality [62,63].

The integration of experimental data from multiple sources has become increasingly sophisticated, with modern knowledge graphs incorporating structural information from x-ray crystallography, nuclear magnetic resonance spectroscopy, and cryo-electron microscopy. This experimental data is further enriched by predicted structural features from AI models, creating a comprehensive representation of protein properties. The resulting graphs maintain provenance information for each data point, enabling researchers to assess the reliability of different information sources and make informed decisions during the design process [64,65].

The graph schema itself has evolved to capture the hierarchical nature of protein organization. Protein nodes are annotated with comprehensive attribute sets describing sequence characteristics, structural features, and functional properties. These attributes are dynamically updated as new information becomes available, ensuring that the knowledge graph remains current. Interaction edges carry detailed metadata including confidence scores, experimental validation methods, and relevant environmental conditions under which the interactions were observed. The schema also captures hierarchical relationships between proteins, domains, and motifs, enabling multiscale analysis of protein properties [66–68].

Advanced reasoning mechanisms have been developed to exploit this rich knowledge representation. Path-based inference algorithms traverse the graph to predict novel protein–protein interactions, utilizing both direct and indirect relationships to generate predictions. These algorithms employ sophisticated path scoring mechanisms that account for the reliability and relevance of different relationship types. Subgraph matching algorithms have been optimized to identify functional modules within the larger protein interaction network, enabling the discovery of conserved structural and functional motifs that can be applied in protein design [64,69,70].

Embedding-based reasoning directly integrates knowledge graphs into generative protein design workflows. By projecting functional ontologies, catalytic mechanisms, and protein–protein interaction networks into high-dimensional vector spaces, knowledge graphs generate functional constraints. These embeddings serve as conditioning tags for autoregressive protein language models, directing the generation of sequences toward specific subcellular localizations, target binding affinities, or enzymatic classes without requiring explicit 3D structural templates [71,72].

### Integration of molecular dynamics and AI

The convergence of molecular dynamics simulations and AI has led to transformative advances in protein design methodology [73]. Enhanced sampling techniques have emerged as a particular focus of innovation, with modern approaches achieving unprecedented efficiency in exploring protein conformational spaces. Neural network-based collective variables have revolutionized enhanced sampling by automatically identifying relevant degrees of freedom for protein motion. These learned variables adapt dynamically during simulations, enabling efficient exploration of complex conformational landscapes that would be difficult to sample using traditional approaches [22,74].

Online learning of bias potentials demonstrates another important advancement in the field. Modern systems continuously update their understanding of the protein’s energy landscape during simulation, allowing for adaptive modification of sampling strategies. This approach has proven particularly effective for studying protein folding pathways and conformational transitions, providing insights that directly inform the design process. The integration of AI-guided adaptive sampling strategies has further enhanced simulation efficiency by focusing computational resources on promising regions of conformational space [75,76].

Force field development has been transformed by the integration of AI techniques. Machine learning potentials trained on high-level quantum mechanical calculations now provide unprecedented accuracy in modeling protein energetics. These potentials capture subtle electronic effects that are difficult to illustrate in traditional force fields, enabling more accurate prediction of protein properties. Neural network corrections to traditional force fields have emerged as a practical approach to improving accuracy while maintaining computational efficiency. These corrections learn to predict and compensate for systematic errors in classical force fields, resulting in more reliable simulations [77,78].

The development of end-to-end differentiable force fields represents a particularly exciting advance. These force fields enable the gradient-based optimization of protein properties, allowing direct optimization of sequence and structure to achieve desired characteristics. The differentiable nature of these force fields facilitates integration with modern deep learning frameworks, enabling seamless combination of physical modeling with machine learning approaches. This integration has proven particularly valuable for designing proteins with specific dynamic properties, as it allows explicit optimization of conformational behavior [79,80].

## AI-Driven Protein Design Tools

The evolution of AI tools for protein engineering is marked by a crucial transition from structure prediction to protein design. Foundational tools like AlphaFold2 solved the long-standing challenge of predicting a protein’s 3D structure from its amino acid sequence. These models are fundamentally discriminative. The field now utilizes generative models, which address the inverse problem: generating a novel protein sequence and backbone to match a desired function or property. Tools like ProteinMPNN and RFdiffusion facilitate this generative approach [30,81].

The landscape of AI-driven protein design tools has been transformed by several breakthrough platforms, each contributing unique capabilities and methodological innovations. As shown in Table 3, commercial AI platforms such as AlphaFold2, RoseTTAFold, and ESM-2 represent the cornerstone of current protein design technology, with widespread applications across pharmaceutical, biotechnology, and academic sectors. These tools symbolize the practical implementation of theoretical advances in AI technology, enabling unprecedented accuracy and efficiency in protein design and analysis.

**Table 3.** Commercial AI platforms for protein design

Platform	Core technology	Commercial applications	Benchmark performance	Key limitations
AlphaFold2 [118–121]	Evolutionary attention	Insilico Medicine: Drug discovery for fibrosis targets; AbCellera: Antibody engineering for therapeutic development	Median backbone root-mean-square deviation (RMSD) of 0.96 Å on CASP14 domains	Static structure prediction; no dynamics
RoseTTAFold [122–124]	Three-track architecture	Outpace Bio: Protein therapeutics design platform; Arzeda: Computational enzyme design for sustainable materials	Median TM-score > 0.8 for monomeric prediction targets; interface prediction accuracy comparable to physical docking	High computational cost; limited to single-chain proteins
ESM-2 [89,125–127]	Protein language model	Absci: AI-driven antibody design and optimization; generate biomedicines: de novo protein design via Chroma platform	Secondary structure prediction accuracy > 85%; contact prediction precision > 70%	Limited physical constraints; generated sequences require extensive experimental screening to confirm folding and function

AI, artificial intelligence; TM-score; Template Modeling score; CASP, Critical Assessment of Structure Prediction

### AlphaFold2 architecture and implementation

AlphaFold2 represents a substantial shift in protein structure prediction. The model requires specific inputs: a target amino acid sequence, multiple sequence alignments generated via database search, and homologous structural templates. It processes these inputs through specialized Evoformer blocks to output static 3-dimensional atomic coordinates alongside a per-residue confidence metric (predicted local distance difference test [pLDDT]). While AlphaFold2 achieves high accuracy for single-domain static structures (frequently exceeding a TM-score of 0.9 on CASP14 targets), it possesses strict methodological boundaries [82]. The model outputs single static conformations, lacking the capability to autonomously simulate protein dynamics, allosteric shifts, or folding pathways. Furthermore, its predictions do not guarantee experimental viability; sequences predicted to fold in silico often require iterative human-in-the-loop refinement to optimize solubility and expression *in vitro* [83,84].

AlphaFold2 performance is categorized by target difficulty: It achieves median backbone root-mean-square deviation values < 1.0 Å for template-based modeling targets, while maintaining high accuracy on challenging free modeling targets that lack homologous templates. While standard inference generates static coordinates, the model outputs specific confidence metrics—the pLDDT and predicted aligned error. Researchers increasingly utilize low pLDDT scores as predictors of intrinsically disordered regions and predicted aligned error matrices to sample conformational ensembles and domain flexibility [83,85]. Performance metrics for AlphaFold2 demonstrate its exceptional capabilities. The system consistently achieves GDT-TS scores exceeding 90 for many protein targets in the CASP14 competition, a performance level that is particularly impressive for single-domain proteins where TM-scores frequently exceed 0.9. The model’s accuracy extends to challenging cases, including the prediction of disordered regions, which had traditionally been difficult to characterize computationally. This performance level has transformed structural biology, enabling reliable structure prediction for previously intractable protein targets [86].

The implementation architecture of AlphaFold2 incorporates several technical innovations that contribute to its exceptional performance. The attention mechanisms are carefully designed to process both local and global structural features simultaneously, while the template processing system can effectively utilize partial structural information when available. The model’s training process incorporates sophisticated loss functions that balance different aspects of structural accuracy, ensuring comprehensive optimization of prediction quality [23].

### RoseTTAFold technical implementation

RoseTTAFold introduces a novel 3-track architecture that processes protein information at multiple levels of abstraction simultaneously. The system implements parallel processing streams for 1-dimensional sequence data, 2-dimensional distance maps, and 3-dimensional coordinate information. This multitrack approach enables the model to capture different aspects of protein structure at various scales, facilitating more accurate predictions and design capabilities [87]. As shown in Table 3, RoseTTAFold has been successfully commercialized through platforms like Outpace Bio for protein therapeutics design and Arzeda for computational enzyme design for sustainable materials, demonstrating its versatility across different industrial applications.

The architecture’s technical implementation includes specialized attention mechanisms for each track, enabling efficient information exchange between different representations. The gradient-based optimization of backbone coordinates allows for precise structural refinement, while the integration of evolutionary information helps guide the prediction process. This sophisticated implementation has enabled RoseTTAFold to achieve remarkable success in various applications, from complex structure prediction to protein–protein interface design and de novo protein creation [88].

### ESM-2 system architecture

Evolutionary-scale prediction (ESM-2) represents the state-of-the-art in protein language models, implementing a sophisticated 33-layer transformer architecture with scaling up to 15 billion parameters. While the 650-million-parameter variant is frequently deployed for standard local inference, the 15-billion-parameter model captures deeper evolutionary covariance patterns. The system incorporates specialized positional encodings designed specifically for protein sequences, enabling better capture of local structural patterns and long-range interactions. The implementation includes multiple attention heads that process different aspects of sequence patterns, allowing the model to simultaneously capture various types of biological relationships [89]. As detailed in Table 3, ESM-2 technology has been leveraged commercially by companies such as Absci for AI-driven antibody design and Generate Biomedicines for de novo protein design through their Chroma platform, illustrating its powerful capabilities in therapeutic protein engineering.

The model demonstrates exceptional performance across multiple prediction tasks. Secondary structure prediction accuracy consistently exceeds 85%, while contact prediction precision surpasses 70% for many protein targets. Perhaps most impressively, the system achieves correlation coefficients above 0.7 in mutation effect prediction tasks without specific training on mutation data, demonstrating strong transfer learning capabilities [90].

### Comprehensive ecosystem of AI tools

The field of protein design is driven by a combination of core platforms and specialized tools, each addressing different challenges in the design process. Table 3 shows the commercial AI platforms that currently dominate the market, which have demonstrated practical application in the pharmaceutical, biotechnology, and industrial sectors. From AlphaFold2 to ESM-2, these technologies have revolutionized structural prediction and sequence–function modeling possibilities through innovative architectural approaches.

Complementing these major business platforms is a range of tools that specialize in specific problems. These new tools complement the limitations of the base platform and focus on interface design, sequence optimization, and segmentation in industrial applications. This diverse technology ecology reflects the complexity of protein engineering and the need for a diverse approach to various design requirements. Table 4 provides a comprehensive overview of these professional tools, detailing their technical underpinnings, major areas of application, and practical examples that demonstrate their scientific and commercial value.

**Table 4.** Overview of additional AI-driven protein design tools

Tool	Developer	Core technology	Key features	Primary applications
ProteinMPNN [30]	Baker Lab	Message passing neural network; native sequence recovery rate of ~52% (outperforming Rosetta by ~20%); highly efficient sequence generation (<1 s/protein)	Multiple sequence design; backbone conditioning; fast sampling	Interface design; sequence optimization
OmegaFold [128]	HeliXon	Single-sequence processing (no MSA requirement); high-resolution prediction achieving TM-scores > 0.7 on orphan proteins; fast inference times	No MSA requirement; fast runtime; attention mechanism	Rapid structure prediction; drug discovery
ProGen2 [129]	Salesforce	Autoregressive generation; trained on >1 billion sequences; capable of generating biochemically viable sequences (up to 400 amino acids) via zero-shot prediction	Controlled synthesis; family-specific design; transfer learning	De novo protein design; function engineering
EvoBind [130]		Physics-guided deep learning integration; outperforms traditional physical docking in specific peptide-binder scenarios; accelerates binder design sampling	Monte Carlo sampling; physics-based scoring; fast prediction	Drug design; protein–ligand interactions
ColabFold [131]	Steinegger Lab	AlphaFold integration	Cloud computing; MMseqs2 search; user-friendly interface	Academic research; educational use
ESM-IF1 [132]	Meta AI	Structure conditioning; high sequence recovery rates across diverse backbone topologies; robust transfer learning capabilities	Structure conditioning; fast sampling; diverse outputs	Structure-based design; sequence generation
trRosetta [133]	Baker Lab	Deep residual networks	Distance prediction; orientation modeling; MSA processing	Structure prediction; contact prediction
ProtGPT2 [134]	OpenBioML	GPT architecture	Zero-shot prediction; sequence generation; domain awareness	Novel protein generation; function prediction

AI, artificial intelligence; MSA, multiple sequence alignment; TM-score, Template Modeling score; GPT, Generative Pre-trained Transformer

## Applications and Impact

The integration of AI in protein design has facilitated transformative advances in fields ranging from therapeutic development and food engineering to industrial biotechnology [91]. These advances, coupled with substantial market growth and commercial success, represent fundamental shifts in our ability to engineer proteins with precise specifications and novel functions. The global impact of these technologies is reflected in both their scientific achievements and their rapidly expanding market presence.

### Therapeutic protein development

The application of AI in therapeutic protein development alters the traditional design-build-test-learn cycle. By enabling rapid in silico screening and structural prediction, AI methods optimize antibody engineering and enzyme replacement therapies prior to resource-intensive clinical evaluation. A landmark achievement in this field was the development of antibody nanocages through AI-driven design [12]. These structures demonstrate a new paradigm in therapeutic delivery, where antibodies are precisely arranged in geometric configurations to enhance their therapeutic efficacy. The design process integrated deep learning models for structure prediction with optimization algorithms that considered both geometric constraints and biological functionality. The resulting nanocages demonstrated superior drug delivery properties compared to traditional antibody formulations, with improved tissue penetration and reduced off-target effects. Commercial implementation of this technology has attracted substantial investment, with major pharmaceutical companies committing substantial resources to AI-driven antibody design platforms. As shown in Table 1, in the last quarter of 2023 alone, 4 major partnerships were formed with potential milestone payments totaling USD 2.2 billion [15,16].

The development of protein-based vaccines expresses another area of substantial impact, particularly highlighted during the COVID-19 pandemic. AI methods have accelerated vaccine development through multiple approaches, including the identification of viral antigenic epitopes, exploration of neutralizing antibodies, and optimization of vaccine design using reverse vaccinology techniques. For example, the Harvard T.H. Chan School of Public Health and the Human Vaccines Project launched the Human Immunomics Initiative, which utilizes state-of-the-art AI models to accelerate vaccine development by analyzing epidemiology, immune monitoring, and network biology data [92]. During the COVID-19 pandemic, AI platforms demonstrated their value in vaccine development through initiatives such as Magar et al.’s machine learning model for antibody exploration, which successfully identified 8 potential antibodies against COVID-19 by analyzing over 1,900 previously reported antigen-antibody sequences. Companies across the pharmaceutical industry have recognized this potential, leading to increased adoption of AI technologies in vaccine development workflows [93].

### Food protein engineering

The food protein field has made important progress through AI-driven protein design, particularly in enhancing protein functionality and developing alternative protein sources [94,95]. Driven by the growing demand for sustainable and nutritious protein solutions, the global food protein market is expected to reach over USD 2.1 trillion by 2030 [96]. Facing the increasing protein demand and higher consumer expectations for food quality, traditional protein improvement methods can no longer meet the requirements.

AI technology has demonstrated important advantages in food protein development in recent years. Amai Proteins from Israel developed a computational platform named Agile Integrated Computational Protein Design (AI-CPD). This method analyzes structural properties of extremophilic proteins to optimize monellin, a natural sweet protein originally identified in tropical berries. By integrating AI-driven amino acid sequence modification with structural prediction algorithms, the team engineered the thermostable sweet protein sweelin. This protein can reduce 40% to 70% of sugar content without affecting taste. Compared to the original protein that denatures at 45 °C, sweelin remains stable even at high temperatures. The product has been tested in various foods such as tomato sauce and chocolate, with professional tasters unable to detect differences. It has received Generally Recognized As Safe certification and is now collaborating with multiple food manufacturers [97].

While early alternative protein development, such as the identification of leghemoglobin by Impossible Foods, relied heavily on traditional bioinformatics and machine learning-assisted screening of natural variants to optimize microbial fermentation, modern food protein engineering increasingly utilizes generative AI [98,99]. Unlike screening-based methods that search existing databases, current generative models enable the de novo design of thermostable or enhanced-flavor proteins that do not exist in nature, representing a shift from optimization to automated synthesis [100].

### Industrial applications

The industrial sector has witnessed substantial advances in biocatalysis through AI and machine learning-driven protein engineering. Over the last 2 decades, biocatalysis has secured its position as a standard approach in the chemist’s toolbox, particularly in manufacturing active pharmaceutical ingredients, as well as fine and bulk chemicals. The integration of computational tools, especially machine learning algorithms, has revolutionized protein engineering for biocatalytic applications and accelerated the development timelines previously needed to optimize enzymes to more efficient variants [101]. The demand and trade for industrial enzymes has been growing steadily. The global enzyme market was valued at USD 8.63 billion in 2019 and is projected to reach USD 14.5 billion by 2027 [102].

A key development has been the application of machine learning methods to enzyme engineering for industrial applications. While traditional enzyme engineering faced limitations in simultaneously improving multiple properties like activity, selectivity, stability, and solvent tolerance, machine learning approaches have enabled more efficient optimization of these multiple parameters. These advances have particularly benefited pharmaceutical manufacturing, where the combination of bioinformatics and machine learning algorithms offers new tools to potentially locate advanced enzyme engineering starting points and more direct routes to de novo and highly engineered high-performance biocatalysts [101].

The field of biocatalysis has seen remarkable advances in the design of enzymes for industrial synthesis. Recent collaborations between major companies demonstrate the practical impact of AI-driven protein engineering. For example, Unilever, working with Arzeda, developed new stain-fighting enzymes for cleaning and laundry products with 50% fewer ingredients while increasing stability, performance, and sustainability. What is particularly notable is the dramatically reduced development time—this enzyme development was achieved in just 18 months, which is 5 times faster than previously possible [103].

In another case, BASF created an AI system called Emollient Maestro that uses machine learning to identify optimal emollient mixes for cosmetic products based on various parameters like sensory properties, performance, and sustainability. This system leverages a huge database of previous experiments together with prediction models to accelerate the prototyping and design of new emollient products [104].

### Future market trajectory and implementation challenges

The protein engineering market exhibits a strong growth trajectory, with projections estimating a valuation of USD 20.86 billion by 2034. However, 10-year market forecasts remain highly speculative. Realizing this projected growth is strictly contingent upon overcoming current technical bottlenecks, specifically the low *in vitro* validation rates of AI-generated designs and the high costs associated with scaling automated wet-lab validation pipelines. Currently, over 95% of commercial protein engineering still relies on traditional screening-based methods [2].

Implementation challenges remain substantial, primarily in production costs and computing resource requirements. While theoretical protein design space is vast—a single 100-amino-acid protein can have 20^100^ sequence variations compared to nature’s 10^12^ explored proteins—practical limitations in production and testing continue to constrain commercial applications. Recent advances in cloud computing infrastructure and improved design tools suggest that these barriers are beginning to lower. The convergence of more accessible computational resources with refined production processes indicates that the field is positioned for accelerated growth and broader commercial adoption in the coming years [2].

As a leading user of AI protein design methods, the pharmaceutical industry provides an insightful case study of the potential and present drawbacks of these methods. Although agreements with potential milestone payments surpassing USD 2.2 billion are displayed in Table 1, there is still little real integration of AI technologies into pharmaceutical research and development workflows. Pharmaceutical companies are adopting AI protein design technologies cautiously, as evidenced by recent industry trends that show that they mainly use these technologies through strategic partnerships rather than acquisitions [105].

A notable variation (a “gap”) exists between what AI and computer models anticipate will work in protein design and what works in lab tests. When scientists design and test proteins in lab settings or living organisms, the success rates are lower than what the computational models predicted. In addition, information asymmetry makes collaboration much more challenging because pharmaceutical businesses with large historical data must weigh knowledge sharing against intellectual property concerns. Furthermore, few companies have fully mastered the complex process of creating proteins, which necessitates the seamless fusion of computational power with pharmacological and biological domain knowledge.

## Challenges and Future Directions

### Technical challenges

One fundamental challenge lies in the accurate prediction of protein dynamics and conformational flexibility [106]. While current methods excel at predicting static structures, proteins are inherently dynamic molecules, and their function often depends on specific conformational changes. Developing AI models that can accurately capture and predict these dynamics represents a crucial next step in the field [107]. This challenge is particularly evident in cases involving allosteric regulation, where conformational changes at sites distant from the active site modulate protein function. Current AI methods struggle to capture these complex dynamic relationships, especially when multiple conformational states are involved in the protein’s functional cycle.

The computational complexity of modeling protein dynamics presents additional challenges, particularly for large proteins or protein complexes. The vast configurational space that needs to be explored, combined with the long time scales of many biological processes, makes it computationally prohibitive to simulate protein dynamics comprehensively. This limitation is exacerbated when considering ensemble-based properties or when multiple dynamic processes occur simultaneously at different time scales. The development of efficient sampling methods that can adequately explore this vast conformational space while maintaining accuracy remains a critical challenge.

The integration of multiple scales of protein behavior presents another big challenge. Proteins function across multiple temporal and spatial scales, from rapid atomic vibrations to slow conformational changes, and from local interactions to cellular-level organization. Current AI methods typically focus on 1 or 2 of these scales, but developing models that can seamlessly integrate across all relevant scales remains a big challenge [108]. This multiscale integration becomes particularly complex when considering protein–protein interactions, where both local contact interfaces and global conformational changes play crucial roles. The challenge extends to understanding how atomic-level perturbations propagate through protein structures to affect macroscopic properties, a key consideration in protein engineering.

The accuracy of current AI models in predicting specific protein properties presents unique challenges. While models have achieved remarkable success in predicting protein structures, accurately predicting properties such as binding affinity, catalytic activity, and stability remains difficult. This challenge is compounded by the fact that these properties often depend on subtle, atomic-level details that current models may not capture effectively. The prediction of protein–protein interaction specificity and the design of selective binding interfaces represent particularly challenging areas where current methods often fall short of desired accuracy levels.

Data quality and availability continue to present challenges, particularly for specialized applications. While the quantity of available protein sequence data has grown exponentially, high-quality structural and functional data remain relatively scarce. This limitation is particularly acute for certain classes of proteins, such as membrane proteins and intrinsically disordered proteins, where experimental characterization is especially challenging [109]. The scarcity of high-quality training data for specific protein classes or functions can lead to biased or incomplete models that may not generalize well to novel design challenges. Furthermore, the available experimental data often contains inherent noise and uncertainties that can affect model training and validation.

Model interpretability expresses another important technical challenge in AI-driven protein design. While deep learning models have demonstrated impressive predictive capabilities, understanding the basis for their predictions remains difficult. This lack of interpretability can make it challenging to identify potential failure modes or to improve model performance. The challenge is particularly acute when models make unexpected predictions, as it can be difficult to determine whether these predictions represent genuine insights or artifacts of the model’s training data and architecture.

A persistent limitation in de novo protein design is the discrepancy between computational prediction and *in vitro* experimental success. While discriminative structure prediction models routinely achieve high accuracy (e.g., median backbone root-mean-square deviation < 1.0 Å for single domains), generative models frequently experience “hallucination” issues [22]. In these instances, models generate sequences that appear structurally plausible in silico but fail to fold, aggregate, or lack stability when expressed *in vitro*. Currently, the experimental success rate for de novo designed functional proteins often falls below 20%, necessitating extensive high-throughput screening to isolate viable candidates [81]. Bridging the validation gap requires integrating physics-based energy scoring to filter AI-generated sequences before experimental synthesis. Successful protein design relies on experimental validation, specifically confirming expression, proper folding, solubility, thermal stability, and target functional activity. Computational models cannot currently account for the complexities of cellular expression systems or aggregation propensity *in vitro*. Consequently, practical workflows require hybrid approaches that anchor AI generation to experimental feedback loops.

Recent frameworks illustrate this necessary integration. For instance, AlphaDesign utilizes AlphaFold not merely as a predictive end point but as a differentiable structural module within a de novo design pipeline, directly linking sequence generation to predicted structural viability [110]. Similarly, approaches integrating platforms like SAGE-Prot emphasize that pure computational generation must be coupled with high-throughput laboratory screening to isolate functional variants [111]. These hybrid paradigms demonstrate that AI currently serves as a highly efficient hypothesis generator rather than an autonomous design solution.

Empirical validation workflows reveal distinct failure modes for AI-designed proteins. While computational metrics (e.g., in silico sequence recovery > 50%) indicate algorithmic success, wet-lab validation frequently exposes aggregation, poor in vivo expression yields, or mispacked hydrophobic cores. Comprehensive experimental validation requires rigorous biophysical characterization: Circular dichroism to confirm secondary structure content, size exclusion chromatography to verify monomeric state and assess aggregation, and x-ray crystallography or cryo-electron microscopy for high-resolution tertiary structural validation. Currently, even top-tier de novo enzyme designs typically require subsequent rounds of laboratory directed evolution to achieve catalytic turnover rates (*k*_cat_) comparable to natural enzymes [81,112].

Despite these remarkable achievements, several key challenges remain in AI-driven protein design. These challenges present opportunities for future development and highlight areas requiring focused research attention.

### Future directions

The future of AI-driven protein design holds immense promise, with several emerging trends likely to shape the field’s development. These advances will likely come from both technological improvements and novel applications of existing methods.

### Advanced AI architectures

The next generation of AI architectures for protein design is likely to address current limitations while introducing new capabilities. One promising direction is the development of multiscale transformers that can simultaneously process information at different levels of protein organization [113]. These models would integrate atomic-level details with higher-order structural features, enabling more comprehensive protein design strategies. Current implementations often struggle with the simultaneous consideration of local interactions and global protein architecture, but emerging research suggests that novel attention mechanisms could bridge this gap, particularly in modeling long-range interactions that influence protein stability and function [13].

Key advances are also expected in the area of generative models. Current approaches often struggle with the precise control of multiple protein properties simultaneously. Future models may incorporate improved conditioning mechanisms that allow designers to specify multiple desired properties with greater precision, such as stability, binding affinity, and catalytic activity [114]. This could enable the design of proteins with complex combinations of structural, functional, and dynamic properties, moving beyond the current limitations of single-property optimization.

The integration of physics-based knowledge with deep learning represents another crucial frontier. While current models learn primarily from data, future approaches may combine the strengths of physical modeling with the pattern-recognition capabilities of AI. This hybrid approach could be particularly valuable for designing proteins to perform novel functions where limited experimental data exists [91,115].

### Experimental integration

The future of protein design will likely see closer integration between computational and experimental methods [116]. The development of high-throughput characterization methods, combined with AI-driven experimental design, could create powerful feedback loops that accelerate the design process.

One promising direction is the development of active learning frameworks that can intelligently guide experimental efforts [117]. These frameworks would identify the most informative protein variants to test, optimizing the use of experimental resources while maximizing knowledge gain. By incorporating experimental feedback in real time, such systems could continuously refine their predictions and design strategies, leading to more accurate and reliable protein designs.

The integration of AI with automated experimental platforms expresses another exciting frontier. Recent advances in laboratory automation, combined with AI-driven design and analysis, could enable fully automated protein engineering pipelines. Such systems could dramatically accelerate the design-build-test cycle, enabling more rapid development of novel proteins.

### Multimodal modeling and regulatory implications

The field is advancing from single-chain protein prediction to multimodal biomolecular modeling. Architectures such as AlphaFold 3 [20] and RoseTTAFold All-Atom [85] enable the simultaneous codesign of proteins, nucleic acids, small-molecule ligands, and posttranslational modifications. These capabilities address critical pharmaceutical bottlenecks in hit-to-lead optimization. Concurrently, the transition of AI-designed biologics into clinical pipelines introduces regulatory challenges. Agencies such as the Food and Drug Administration require strict reproducibility and interpretability. Establishing standardized computational benchmarks and mandated wet-lab validation criteria will be essential to translate in silico predictions into approved therapeutics.

## Conclusion

The integration of AI in protein design has begun to fundamentally transformed our ability to engineer proteins with precise specifications and novel functions. This review has highlighted the remarkable progress achieved by applying deep learning, protein language models, and integrated design approaches. These advances have enabled unprecedented achievements across multiple domains, from therapeutic development to environmental applications.

The field continues to evolve rapidly, with new methods and applications emerging regularly. The challenges that remain, particularly in areas such as protein dynamics and multiscale integration, depict opportunities for future development. The increasing integration of AI with experimental methods and the emergence of new application areas suggest that the impact of AI on protein design will continue to grow.

Looking forward, we anticipate that AI-driven protein design will play an increasingly central role in addressing major societal challenges, from healthcare to environmental protection. The combination of advancing AI technologies, improving experimental methods, and expanding application domains suggests that we are still in the early stages of realizing the full potential of this transformative approach to protein engineering.

As we move forward, continued advancement will require close collaboration between computational and experimental scientists, as well as engagement with emerging technologies such as quantum computing and automated experimentation. The future of protein design lies in successfully integrating these various approaches, working together to unlock new possibilities in protein engineering and its applications.
